# Fu brick tea alleviates high fat induced non-alcoholic fatty liver disease by remodeling the gut microbiota and liver metabolism

**DOI:** 10.3389/fnut.2022.1062323

**Published:** 2022-12-22

**Authors:** Yan Tang, Bowei Chen, Xin Huang, Xu He, Jian Yi, Hong Zhao, Fengming Tian, Yingfei Liu, Baiyan Liu

**Affiliations:** ^1^Department of Basic Medicine, Yiyang Medical College, Yiyang, China; ^2^The First Hospital, Hunan University of Chinese Medicine, Changsha, China; ^3^MOE Key Laboratory of Research and Translation on Prevention and Treatment of Major Diseases in Internal Medicine of Traditional Chinese Medicine, Hunan University of Chinese Medicine, Changsha, China; ^4^Department of Clinical Medicine, Yiyang Medical College, Yiyang, China; ^5^Hunan Academy of Chinese Medicine, Changsha, China

**Keywords:** fu brick tea, lipids, metabolomics, non-alcoholic fatty liver disease, gut microbiota

## Abstract

Fu brick tea (FBT) and its extracts have good lipid-lowering effects and have been used in the treatment of obesity in previous studies. Unfortunately, the therapeutic effect of FBT on non-alcoholic fatty liver disease (NAFLD) has not been thoroughly studied. In this study, we explored the mechanism by which FBT alleviates NAFLD from the perspective of the gut microbiota and liver metabolites. The results showed that FBT could reduce the body weight, liver weight and abdominal fat of NAFLD mice, and improve liver pathological morphology, liver lipid deposition, blood lipids and liver function. Moreover, FBT improved the diversity of the gut microbiota and changed the profile of liver metabolism in NAFLD mice. Further studies showed that FBT could ameliorate the cecum barrier, and regulate the effects of factors related to lipid synthesis in the cecum and liver of NAFLD mice. In conclusion, the present study confirmed that FBT can alleviate high fat induced NAFLD by regulating the homeostasis of the gut microbiota and liver metabolites.

## Graphical Abstract

Fu brick tea (FBT’s) working probably mechanisms on NAFLD. 
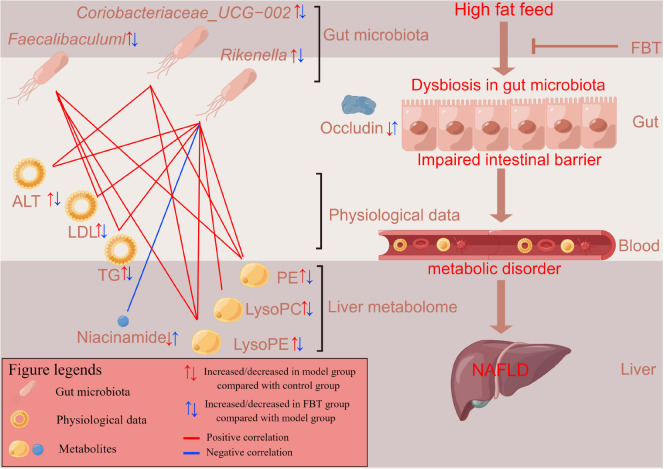

## 1 Introduction

With the changes in people’s dietary structure and lifestyle, the incidence of non-alcoholic fatty liver disease (NAFLD) is on the rise (1). Studies have shown that the prevalence of NAFLD in adults is 17–33%, which has become the second largest liver disease after viral hepatitis, especially in obese and overweight people. If the disease continues to develop, such as leading to repeated degeneration and necrosis of hepatocytes, it will eventually turn into liver cirrhosis or even hepatocellular carcinoma (2). At present, there is still a lack of effective drugs to treat NAFLD. Exploring safe and effective methods to prevent or improve NAFLD is a popular research topic.

A large number of clinical studies have shown that the composition of the gut microbiota of NAFLD patients has changed (3–5), and the gut microbiota can play a key role in the progression of NAFLD through the gut microbiota–liver axis (6, 7). The gut microbiota–liver axis refers to the production of different signaling molecules by the microbiota through the gut and bacterial products, which affect the metabolic status of different organs, including the liver (8). It is suggested that the regulation of the gut microbiota may be a new target for the prevention and treatment of NAFLD.

In recent years, research on post-fermented tea with microbial fermentation as the hallmark technology has gradually deepened. Fuzhuan brick tea (FBT), as a kind of post-fermented tea with green tea as a raw material, fermented by *Eurotium cristatum*, belongs to the dark tea category (9). During the fermentation process of FBT, some new are substances formed, such as theaflavin, thearubin, and theabrownin (10). Previous studies have shown that FBT and its extract can reduce blood lipids and oxidative stress in high-fat fed mice, improve lipid metabolism (11–13), and reduce obesity by regulating the gut microbiota (14, 15). However, whether FBT can alleviate NAFLD injury through the gut microbiota has not been reported.

**FIGURE 1:**
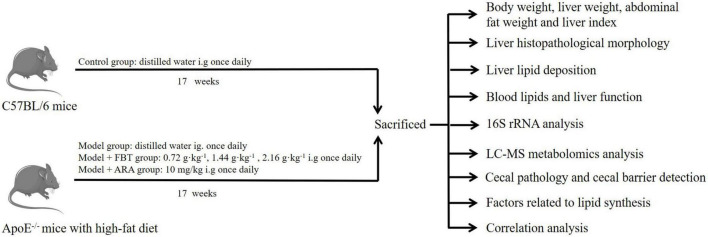
Experimental design.

In this study, we induced a NAFLD model by feeding apolipoprotein E knock out (ApoE^–/–^) mice a high-fat diet and treated them with FBT. We first investigated whether FBT could improve body weight, liver pathological injury, blood lipids and liver function in NAFLD mice. In addition, Illumina high-throughput sequencing technology and metabolomics were used to study the effects of FBT on intestinal microbiome diversity and liver metabolites in NAFLD mice. Moreover, we evaluated the potential effects of FBT on factors related to intestinal and liver lipid metabolism in NAFLD mice. The experimental design is shown in Figure 1. This study revealed the mechanism of FBT in the prevention and treatment of NAFLD from the perspective of the gut microbiota and liver metabonomics for the first time.

## 2 Materials and methods

### 2.1 Animals

Sixty 8-week-old male specific pathogen-free (SPF) ApoE^–/–^ C57BL/6J mice were purchased from Jiangsu GemPharmatech Co., Ltd.^1^ with a body weight of 25 ± 5 g, strain name: ApoE Cas9-Ko, strain number T001458, and license number CXK(SU) 2018-0008. Twelve 8-week-old male wild-type C57BL/6J mice, weighing 25 ± 5 g, were also purchased from GemPharmatech Co., Ltd. The experimental protocol was approved by the ethics committee of the First Affiliated Hospital of Hunan University of Chinese Medicine (ZYFY20210710).

### 2.2 Drugs and reagents

The following were used in this study: 2020 Tianfu tea (Hunan Baishaxi Tea Industry Co., Ltd.); atorvastatin (ARA) (Pfizer Pharmaceutical Co., Ltd., specification: 10 mg, batch number: 110590); alanine aminotransferase (ALT) assay kit and aspartate aminotransferase (AST) assay kit (Nanjing Jiancheng Bioengineering Institute, batch number: C010-2-1); Occludin antibody (Wuhan Proteintech, batch number: 27260-1-AP); Multimeric anti-rabbit IgG-HRP kit(Wuhan Boster, batch number: SV0002); RNA extraction kit (Beijing Beyotime, batch number: R0026); cDNA first strand synthesis Kit (Beijing Beyotime, batch number: D7178); and SYBR Green qPCR mix (Beijing Beyotime, batch number D7265).

### 2.3 Animal feed

High fat feed (batch number: H10141) containing 21% fat and 0.15% cholesterol was provided by Beijing Huafukang Biotechnology Co., Ltd. The specific formula included cholesterol, calcium carbonate, corn oil, anhydrous cream, casein, methionine, maltodextrin, corn starch, cellulose, mineral mixture and vitamin mixture. Other ordinary maintenance feed was produced by Hunan SJD Laboratory Animal Co., Ltd.

### 2.4 Experimental methods

#### 2.4.1 Drug preparation

To ensure the freshness of the FBT, each boiling was enough for 1 week dosage. The specific method was as follows: 20 g of FBT was accurately weighed and fully crushed, wrapped with sterile gauze and tied tightly in a sterile beaker. The tea leaves were equipped with sterile grade III water at a ratio of 1 g:15 mL, and the amount of water added was less than 2–3 cm of the tea bag. After soaking sufficiently for 0.5 h and boiling for 30 min, the tea was taken and placed in another container. Grade III water was added, and boiling was continued. Then, the tea was combined and concentrated to 185 mL (high dose) and centrifuged at 3000 × *g* for 15 min to remove the residue, and the supernatant was taken. The samples were stored in a refrigerator at 4°C.

#### 2.4.2 Animal feeding

Mice were fed in SPF grade animal rooms equipped with individual ventilated cages (IVCs) and fed in isolated cages with four mice per cage. The room temperature was maintained at 20 ± 5°C, with a relative humidity of 40–70% and alternating light and dark cycles. ApoE^–/–^ mice and wild-type mice were fed the same amount of high-fat diet and normal diet, respectively. Animals were allowed free access to drinking water, the bedding was changed every 2 days, and the cages and water bottles were disinfected regularly.

#### 2.4.3 Grouping, modeling, and administration of mice

After adaptive feeding, 60 ApoE^–/–^ mice were randomly divided into the model group, ARA group and FBT high-dose (FBT-H), medium-dose (FBT-M), and low-dose (FBT-L) groups, with 12 mice in each group, and a high fat diet was given daily. The other 12 male wild-type mice were used as the control group and were fed a normal growth diet quantitatively every day. Mice in each group were given drugs by gavage intervention every morning for 17 weeks (16). In addition, the recommended daily consumption of a 60 kg adult in the reference tea absorption test is 10 g (17). According to the body surface area of humans and animals, the high, medium and low doses of FBT were 2.16, 1.44 and 0.72 g⋅kg^–1^⋅d^–1^, respectively. ARA was administered by gavage at a dose of 10 mg⋅kg^–1^⋅d^–1^.

#### 2.4.4 Specimen collection and processing

Mice in each group were fasted for 12 h after the 17th week of intragastric administration. After anesthesia, the eyeballs were removed for blood collection, and the blood was collected in a 1.5 mL EP tube, which was kept at room temperature for 2 h and centrifuged at 12, 000 × *g* at 4°C for 15 min. The serum was collected by pipette and then divided into an EP tube and stored in refrigerator at −80°C. The mice were sacrificed by cervical dislocation. The mice were placed on ice to obtain specimens. The abdominal cavity was opened to expose the liver. The whole liver was quickly cut off with ophthalmic scissors and the abdominal fat was scraped. The liver and abdominal fat were weighed on a precision balance and calculated the liver index. After rinsing with precooled normal saline, the liver tissue and cecal tissue of the same part was cut for pathological examination. In addition, the liver tissue and cecal tissue of the same part were weighed and quickly frozen in liquid nitrogen, and then transferred to −80°C freezer for storage. In addition, the cecal contents were removed under aseptic conditions, quickly flash-frozen in liquid nitrogen, and then transferred to a −80°C freezer for storage.

#### 2.4.5 Hematoxylin and eosin (HE) staining

After the liver and cecum tissue were fixed with 4% paraformaldehyde, they were routinely dehydrated by an automatic dehydrator and then embedded. The tissue wax block was fully cooled and put into a microtome to prepare 4 μm thick slices that were dried on the spreader and put into the oven at 70°C for 1 h. The tissue morphology was observed after conventional HE staining and sealing. Finally, the slices were photographed at 100× and 200× magnification. The pathological severities of steatosis, lobular inflammation, and hepatocyte ballooning were determined using the NAFLD activity score (NAS) as previously described (18).

#### 2.4.6 Oil red O staining

The frozen liver tissue was dehydrated with sucrose solution and embedded with optimal cutting temperature (OCT) sol to make frozen sections with a thickness of 10 μm. Staining was performed using oil red O stain. Finally, the slices were photographed at 100× and 200× magnification. The staining extent of oil red O staining was analyzed and quantified using ImageJ. The integrated optical density (IOD) was then observed. The mean optical density (MOD) was calculated using the following formula: MOD = IOD/sum area (19).

#### 2.4.7 Blood lipid and liver function indexes

Mouse serum was collected and analyzed using an automated biochemical analyzer for ALT, AST, triglyceride (TG), total cholesterol (TC), high-density lipoprotein (HDL) and low-density lipoprotein (LDL) levels.

#### 2.4.8 Gut microbiota analysis

Genomic DNA was extracted from the cecal contents of mice by a DNA extraction kit, and then a NanoDrop2000 was used to detect the concentration of DNA. Using genomic DNA as a template, PCR was performed using Tks Gflex DNA Polymerase (Takara) with BARCODE specific primers according to the selected sequencing region to ensure amplification efficiency and accuracy. The primer sequences were as follows: V3-V4 forward primer: 343F TACGGRAGGCAGCAG, and reverse primer: 798R AGGGTATCTAATCCT.

Subsequently, Qubit quantification was performed on the purified PCR products. Equal amounts of samples were mixed according to the concentration of PCR products and sequenced using Illumina MiSeq PE250. Trimmomatic software was used to remove impurities from the original two terminal sequences. According to the similarity of sequences, the sequences were classified into multiple operational taxonomic units (OTUs) using Vsearch software. The QIIME software package was used to select the representative sequences of each OTU, and all representative sequences were aligned with the database for annotation. Subsequently, the microbial community structure was assessed by alpha diversity analysis and beta diversity analysis. Combining Phylogenetic Investigation of Communities by Reconstruction of Unobserved States (PICRUSt) and 16S functional group prediction was performed in Kyoto Encyclopedia of Genes and Genomes (KEGG) of 16 S rDNA gene sequences. The database construction and sequencing and subsequent data analysis were completed by Shanghai OE Biomedical Technology Co., Ltd.

#### 2.4.9 Metabolomic analysis

The metabolomic study of mouse liver was performed by liquid chromatography-mass spectrometry (LC-MS), and the experimental method was similar to our previous study (20). A total of 100 mg of liver tissue sample was accurately weighed into a 1.5-ml EP tube and 20 μL internal standard and 600 μL of methanol water (v:v = 4:1) were added. Two small steel balls that were precooled in a −20°C freezer for 2 min were added, and then the samples were ground in a grinder (60 Hz, 2 min). Ultrasonic extraction was performed in an ice water bath for 10 min. The samples were allowed to stand at −20°C for 2 h. The samples were then centrifuged for 10 min (13,000 rpm, 4°C), aspirated with a syringe to obtain 150 μL of supernatant, and filtered using a 0.22 μm organic phase pinhole filter. The filtered samples were transferred to LC injection vials and stored at −80°C until LC–MS analysis. The chromatographic and mass spectrometric conditions were the same as before (21). LC-MS analysis was performed by Shanghai OE Biomedical Technology Co., Ltd, and the analysis protocol of metabolomics is shown in Supplementary material 1.

#### 2.4.10 Immunohistochemistry staining

The fixed cecal tissues were taken, dehydrated by an alcohol gradient, embedded in paraffin, and cut into 5 μm thick sections according to a previously reported method (22). 3% hydrogen peroxide for 10 min, citrate buffer microwave radiation antigen repair, confining liquid closed. Occludin (1:800) antibody was added and incubated overnight at 4°C. The secondary antibody was added and incubated at 37°C for 30 min. After DAB chromogenic agents were added, hematoxylin was redyed, ethanol was dehydrated, xylene was transparent, and tablets were sealed. Finally, the slices were photographed at 100× and 200× magnification. IOD based ImageJ was used to analyze and quantify the ratio of the positive expression area to the total area.

#### 2.4.11 RT-qPCR detection

RT-qPCR was used to detect the mRNA levels of *Occludin*, HMG-CoA reductase (*HMGCR*), peroxisome proliferator-activated receptor γ (*PPAR*-γ) and stearoyl-CoA desaturase 1 (*SCD-1*) in the cecum and liver. Fifty milligrams of tissue was placed in a 1.5-ml centrifuge tube, and 600 μL of precooled lysate was quickly added. The mixture was homogenized with a microelectric homogenizer on ice, and RNA was extracted by the centrifugal column method. The RNA was reverse transcribed into cDNA, followed by PCR amplification. The primer sequences are shown in Table 1. β-*actin* was selected as the reference gene, and the relative mRNA expression was calculated by the 2^–ΔΔ^ ^CT^ method.

**TABLE 1:** PCR primer sequences.

Gene	Primer sequence	Product length (bp)	Melting temperature (°C)
β*-actin*	F: CGTTGA CATCCGTAAAGAC	201	60
	R: TGGAAGGT GGACAGTGAG		
*HMGCR*	F:TGGCAGGAC GCAACCTCTAT	222	60
	R:TGACGGCTTC ACAAACCACA		
*PPAR*-γ	F: GGAGCCTAAG TTTGCTGTG	147	60
	R: TGCAGCAGGTT GTCTTGGATG		
*SCD-1*	F: ACACGCCGACC CTCACAACTC	199	60
	R: CAGTGTGGG CAGGATGAAGCA		
*Occludin*	F: ATAAGTCAA CACCTCTGGTG	96	60
	R: TTACCATTG CTGCTGTACC		
			

### 2.5 Statistical analysis

The measurement data are expressed as the mean plus or minus standard error ($\bar{x}$ ± s). One way analysis of variance (ANOVA) was used for the comparison of multiple groups of data in the experiment, and the least significant difference (LSD) test was used for multiple comparisons. *P* < 0.05 indicates that the difference is statistically significant.

In the analysis of the gut microbiota, Usearch software was used to detect *de novo* chimeras and to cluster the data to obtain OTUs for alpha diversity and beta diversity analysis. Linear discriminant analysis (LDA) was used to estimate the communities or species that had significant differences in sample division.

In metabolomic analysis, variable importance of projection (VIP) > 1 and *P* < 0.05 were used to screen differential metabolites. Metabolic pathway enrichment analysis was performed using the KEGG database.^2^

## 3 Results

### 3.1 Effects of FBT on body weight, liver weight, and abdominal fat of NAFLD mice

**FIGURE 2:**
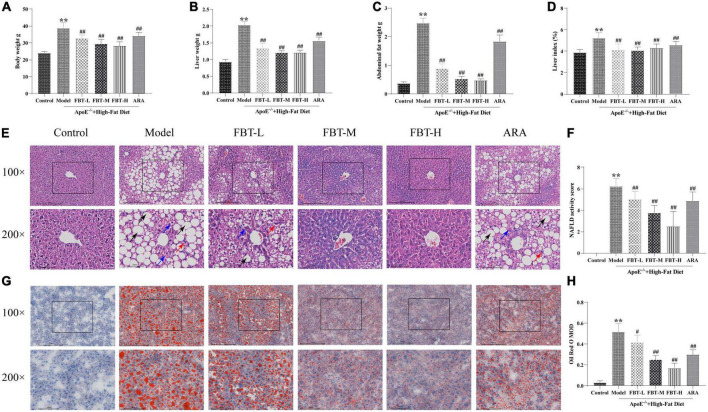
Effects of fu brick tea (FBT) on body weight, liver weight, abdominal fat, histopathological morphology, and lipid deposition of NAFLD mice. **(A)** Body weight was reduced in NAFLD mice after treatment with FBT; **(B)** liver weight was reduced in NAFLD mice after treatment with FBT; **(C)** abdominal fat was reduced in NAFLD mice after treatment with FBT; **(D)** FBT treatment decreased the liver index in NAFLD mice; **(E)** HE staining indicated that FBT treatment ameliorated liver steatosis in NAFLD mice (100× and 200×). Black arrows indicate the steatosis of hepatocytes, blue arrows indicate cytoplasmic rarefaction, and red arrows indicate lobular inflammation; **(F)** FBT treatment decreased the NAS score in NAFLD mice; **(G)** oil red O staining showed that FBT treatment improved the lipid accumulation in the liver; **(H)** the liver lipid contents were decreased in NAFLD mice after FBT treatment. *N* = 12. **P < 0.01 vs. control group. ^#^*P* < 0.05, ^##^*p* < 0.01 vs. model group.

Compared with the control group, the body weight, liver weight, abdominal fat weight and liver index of mice in the model group were significantly increased (*P* < 0.01). Compared with the model group, the body weight, liver weight, abdominal fat weight and liver index of mice in the FBT-L, FBT-M, FBT-H, and ARA groups were decreased (*P* < 0.01), as shown in Figures 2A–D.

### 3.2 Effect of FBT on the histopathological morphology of liver tissue of NAFLD mice

In the control group, the structure of hepatic lobules was intact, and the hepatic cord was radially arranged under low magnification (100×); at high magnification (200×), hepatocytes were structurally intact and uniform in size, with intermediate nuclei, homogenous red-stained cytoplasm, no steatosis, and hepatic sinusoids. In the model group, the structure of the hepatic lobules was disordered under low magnification, and a large number of fat vacuoles appeared, especially in the central lobule area. The fat vacuoles were mainly bullae steatosis, accompanied by a small amount of vesicular steatosis. At high magnification, hepatocytes were disorganized and varied in size. The nuclei were crowded to the edge by vacuoles in hepatocytes, the hepatic sinusoids were unclear, and hepatocyte ballooning and lobular inflammation appeared. Compared with the control group, the NAS score was significantly higher in the model group (*P* < 0.01). At low magnification, the hepatic lobule structure of the FBT-L, FBT-M, and FBT-H groups was improved compared with that of the model group, and the number of fat vacuoles decreased in a dose-dependent manner. At high magnification, the hepatocyte structure and size were improved compared with the model group, and cytosolic, fatty vacuolation, hepatocyte ballooning and lobular inflammation were improved in a dose-dependent manner. In the ARA group, the hepatic lobule structure was disordered under low magnification, accompanied by a large number of macrovesicular and vesicular mixed steatosis. At high magnification, the structure of hepatocytes was disordered, vacuoles of different sizes could be seen in the cells, and hepatocyte ballooning and lobular inflammation appeared. Likewise, the NAS score was significantly lower in the FBT-L, FBT-M, FBT-H, and ARA groups than in the model group (*P* < 0.01), as shown in Figures 2E, F.

### 3.3 Effect of FBT on the lipid deposition in the liver tissue of NAFLD mice

According to the oil red O staining, the nuclei of liver tissue in the control group were blue without orange–red staining. Compared with the control group, the model group had large connected orange staining (*P* < 0.01). Compared with the model group, the orange red staining area of each dose group of FBT decreased in a dose-dependent manner (*P* < 0.01 or 0.05). In addition, orange-red staining could be seen in the ARA group (*P* < 0.01), as shown in Figures 2G, H.

### 3.4 Effects of FBT on blood lipids in NAFLD mice

**FIGURE 3:**
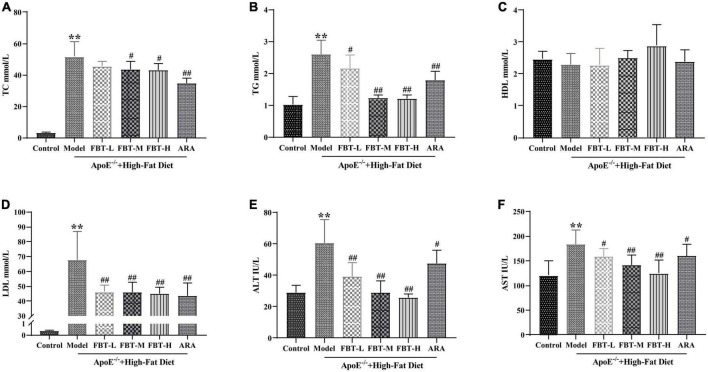
Effects of FBT on blood lipids and liver function of NAFLD mice. **(A)** FBT treatment decreased TC in NAFLD mice; **(B)** FBT treatment decreased TG in NAFLD mice; **(C)** FBT treatment had no significant effect on HDL in NAFLD mice; **(D)** FBT treatment decreased LDL in NAFLD mice; **(E)** FBT treatment decreased ALT in NAFLD mice; **(F)** FBT treatment decreased AST in NAFLD mice. **P < 0.01 vs. control group. *N* = 12. ^#^*P* < 0.05, ^##^*p* < 0.01 vs. model group.

Compared with that in the control group, the TC in the model group was significantly increased (*P* < 0.01); compared with that in the model group, the TC in the FBT-M, FBT-H, and ARA groups was decreased (*P* < 0.01 or 0.05). Compared with the levels in the control group, TG and LDL in the model group were significantly increased (*P* < 0.01); compared with the level in the model group, the TG of the FBT-L, FBT-M, FBT-H, and ARA groups was decreased (*P* < 0.01 or 0.05). In addition, there was no significant difference in HDL among the groups, as shown in Figures 3A–D.

### 3.5 Effect of FBT on liver function in NAFLD mice

Compared with the levels in the control group, the levels of ALT and AST in the model group were significantly increased (*P* < 0.01). Compared with the levels in the model group, the levels of ALT and AST in the FBT-L, FBT-M, FBT-H, and ARA groups were significantly decreased (*P* < 0.01 or 0.05) (Figures 3E, F).

### 3.6 Effect of FBT on the gut microbiota in NAFLD mice

**FIGURE 4:**
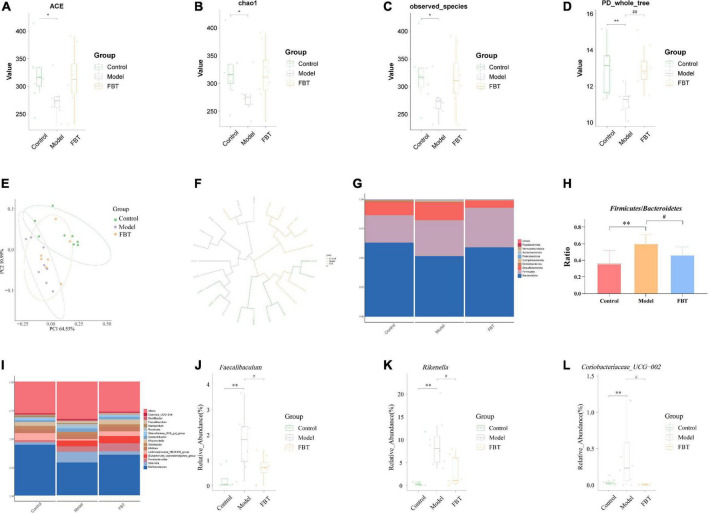
Effects of FBT on gut microbiota diversity and abundance of NAFLD mice. **(A)** ACE index was increased in NAFLD mice after treatment with FBT; **(B)** Chao1 index was increased in NAFLD mice after treatment with FBT; **(C)** observed species index was increased in NAFLD mice after treatment with FBT; **(D)** PD_whole_tree index was increased in NAFLD mice after treatment with FBT; **(E,F)** PCoA and UPGMA showed more similar beta diversity between the FBT group and control groups than that between the model and control group; **(G,H)** at the phylum level, FBT treatment increased the *Bacteroidetes*/*Firmicutes* ratio in NAFLD mice; **(I–L)** at the genus level, FBT treatment increased the abundances of *Faecalibaculum*, *Rikenella*, and *Coriobacteriaceae_UCG-002*. *N* = 8. *P < 0.05, **p < 0.01 vs. control group. ^#^*P* < 0.05, ^##^*p* < 0.01 vs. model group.

We used the ACE index, Chao1 index, observed species index and Shannon index to evaluate the alpha diversity. The results showed that compared with those of the control group, the ACE index, Chao1 index, observed species index and Shannon index of the model group were significantly decreased (*P* < 0.01 or 0.05). Treatment with FBT restored the gut microbiota associated index (*P* > 0.05, compared with the control group). It is suggested that FBT can partially improve the diversity and richness of the gut microbiota in the model group, as shown in Figures 4A–D. In addition, we used the principal coordinates analysis (PCoA) and the unweighted pair group method with arithmetic mean (UPGMA) to evaluate the beta diversity. PCoA based on weighted UniFrac distance showed that the microbial composition and structure of the model group and the control group were significantly different, and the FBT group was similar to the control group, suggesting that FBT can partially restore the composition and structure changes in the gut microbiota in the model group, as shown in Figure 4E. Likewise, UPGMA analysis indicated that the distance from the control group to the FBT group was closer than either to the model group, as shown in Figure 4F.

The community column chart can be used to compare the dominant species and their relative abundances in the gut microbiota in different samples. At the phylum level, the dominant bacteria with differences in the gut microbiota of mice in each group mainly belonged to *Bacteroidota*, *Firmicutes* and *Desulfobacterota*, as shown in Figure 4G. The mice in the model group showed a significant increase in the ratio of *Firmicutes/Bacteroidetes* (*P* < 0.01). In contrast, FBT treatment reversed the ratio of *Firmicutes/Bacteroidetes* (*P* < 0.05), as shown in Figure 4H. At the genus level, *Muribaculaceae* was the dominant flora in each group (Figure 4I), and the model group mice showed an increase in the relative abundance of *Faecalibaculum*, *Rikenella* and *Coriobacteriaceae_UCG-002* (*P* < 0.01). In contrast, FBT decreased the relative abundance of *Faecalibaculum*, *Rikenella* and *Coriobacteriaceae_UCG-002* (*P* < 0.05) in the model group, as shown in Figures 4J–L.

**FIGURE 5:**
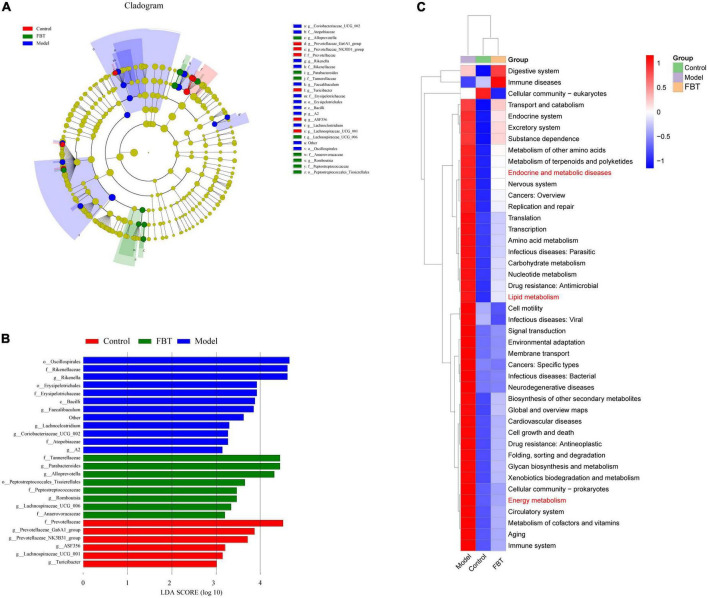
Effects of FBT on gut key microbiota and microbiota biological function of NAFLD mice. **(A,B)** FBT treatment altered key microbiota of the gut in NAFLD mice; **(C)** the differential metabolic pathways of FBT on NAFLD were predicted using PICRUSt analysis.

Linear discriminant analysis effect size (LEfSe) analysis was applied to determine the key microbiota differentially represented in the FBT group. It was found that the key microbiota at the order level was *Eptostreptococcales_Tissierellale*, and at the family level, they were *Tannerellaceae*. At the genus level, the key microbiota was *Parabacteroides*, as shown in Figures 5A, B.

To determine whether changes in the classification of gut microbes affect their functions, we performed functional predictions on representative sequences at the genus level by PICRUSt2. The results showed that compared with those in the control group, the intestinal microbes in the model group had significant differences in endocrine and metabolic diseases, lipid metabolism and energy metabolism. FBT intervention reversed the changes in the above pathways, as shown in Figure 5C.

### 3.7 Effect of FBT on the metabolic profile of the liver in NAFLD mice

**FIGURE 6:**
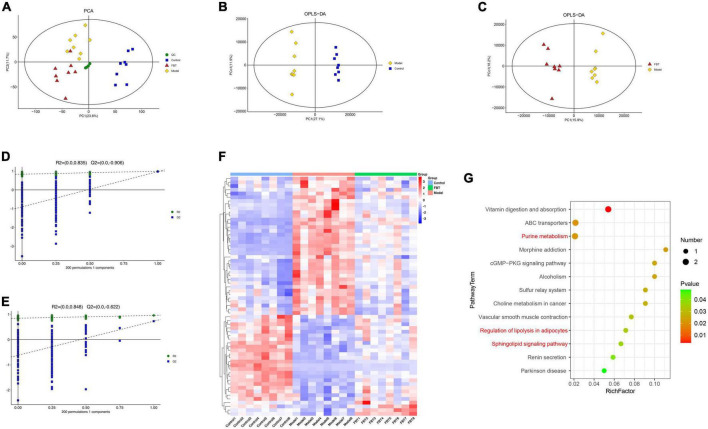
Effects of FBT on the metabolic profile of the liver of NAFLD mice. **(A)** Scores plots of PCA between each group; **(B)** scores plots of OPLS-DA of LC-MS (Model vs. Control); **(C)** scores plots of OPLS-DA of LC-MS (FBT vs. Model); **(D)** permutation of OPLS-DA model (Model vs. Control); **(E)** permutation of OPLS-DA model (FBT vs. Model); **(F)** differentially abundant metabolites between each group; **(G)** analysis of metabolic pathway enrichment about differentially abundant metabolites.

We analyzed the effect of FBT on the metabolic profile of the liver by LC-MS. First, the principal component analysis (PCA) was carried out, and it was found that quality control (QC) samples had a high degree of aggregation, indicating that the method established by the experiment had good repeatability and stability, as shown in Figure 6A. The OPLS-DA showed that there was a significant separation between the groups, suggesting that there were significant differences between the groups, as shown in Figures 6B, C. To prevent overfitting of the model from overfitting, s sevenfold cross validation and 200 response permutation testing (RPT) were used to investigate the quality of the model. The results showed that R2 = 0.835 and Q2 = –0.906 in the model group compared with the control group (Figure 6D). Compared with the model group, the R2 and Q2 of the FBT group were 0.848 and –0.622, respectively (Figure 6E). The above results show that the OPLS-DA model is stable and has good predictive ability. Finally, according to the VIP > 1 and *P* < 0.05, the differential metabolites between different groups were determined. A total of 345 differential metabolites were found between the model group and the control group, and 212 differential metabolites were found between the FBT group and the model group, as shown in Supplementary Table 1.

Further analysis revealed that FBT reversed 64 dysregulated metabolites in the model group. Thirty-seven metabolites were increased in the model group and decreased in the FBT group. Another 27 metabolites were decreased in the model group and increased in the FBT group, as shown in Figure 6F and Supplementary Table 2. The metabolic pathways involved in the above 64 metabolites were analyzed based on the KEGG database. Finally, we found that the regulation of adipocyte lipolysis and the sphingolipid signaling pathway were significant metabolic pathways (*P* < 0.05), as shown in Figure 6G. Interestingly, we found that the significantly enriched pathways by metabolomics, namely, regulation of sphingolipid metabolism and adipocyte lipolysis, were very similar to the metabolic pathways of the gut microbiota predicted by PICRUSt, that is, lipid metabolism and energy metabolism. This finding suggests that the effects of FBT intervention on the gut microbiota and liver metabolites may be relevant.

### 3.8 Effect of FBT on the cecum morphology and intestinal barrier in NAFLD mice

**FIGURE 7:**
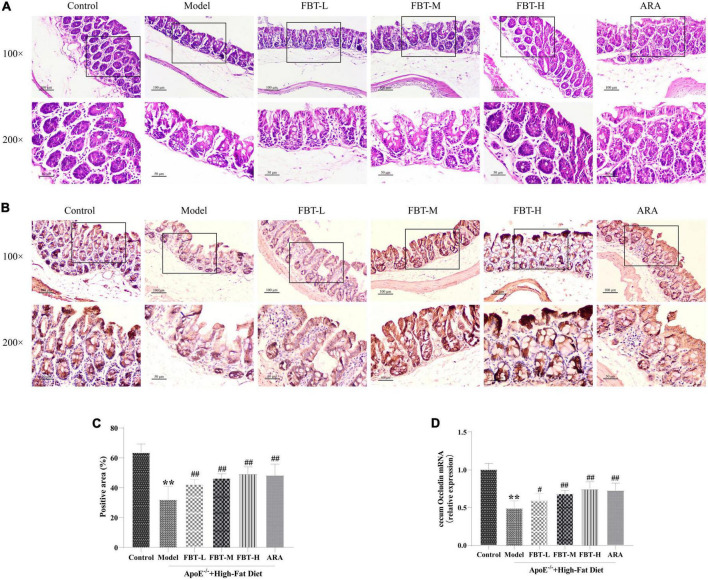
Effects of FBT on cecal pathology and the cecal barrier of NAFLD mice. **(A)** HE staining indicated that FBT treatment ameliorated cecal mucosa in NAFLD mice (100× and 200×); **(B,C)** immunostaining showed that the protein expression of Occludin in the cecum was increased after FBT treatment; **(D)** RT-qPCR showed that the gene expression of *Occludin* in the cecum was increased after FBT treatment. **p < 0.01 vs. control group. *n* = 8. ^#^*p* < 0.05, ^##^*p* < 0.01 vs. model group.

Compared with the control group, the number of intestinal villi in the cecal tissue of mice in the model group was reduced and the intestinal integrity was damaged. Interestingly, intervention with FBT significantly improved these changes, as shown in Figure 7A. In addition, the tight junction protein Occludin is mainly expressed in the lateral apical cytoplasm of epithelial cells, which is an important part of forming tight junctions between intestinal epithelial cells and plays an important role in maintaining the integrity of the intestinal wall. We found that compared with the control group, the protein and mRNA expression of Occludin in the cecum of the model group mice decreased significantly (*P* < 0.01). Compared with the model group, the expression of Occludin protein and mRNA in the cecum of the FBT-L, FBT-M, FBT-H, and ARA groups increased significantly (*P* < 0.01 or 0.05), as shown in Figures 7B–D. These findings suggest that FBT can improve cecal morphology and the intestinal barrier in NAFLD mice.

### 3.9 Effects of FBT on factors related to lipid synthesis in the cecum and liver of NAFLD mice

**FIGURE 8:**
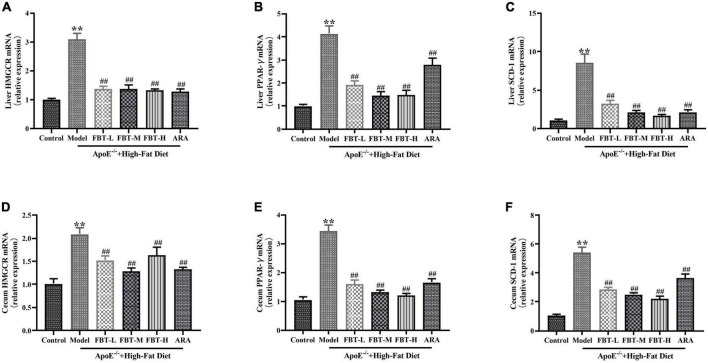
Effects of FBT on factors related to lipid synthesis in the cecum and liver of NAFLD mice. **(A)** HMGCR in the liver was decreased in NAFLD mice after treatment with FBT; **(B)** PPAR-γ in the liver was decreased in NAFLD mice after treatment with FBT; **(C)** SCD-1 in the liver was decreased in NAFLD mice after treatment with FBT; **(D)** HMGCR in the cecum was decreased in NAFLD mice after treatment with FBT; **(E)** PPAR-γ in the cecum was decreased in NAFLD mice after treatment with FBT; **(F)** SCD-1 in the cecum was decreased in NAFLD mice after treatment with FBT. *N* = 12. **P < 0.01 vs. control group. ^##^*P* < 0.01 vs. model group.

Compared with the expression in the control group, the mRNA expression levels of *HMGCR*, *PPAR*-γ and *SCD-1* in the liver of the model group were significantly increased (*P* < 0.01). Compared with the expression in the model group, the mRNA expression levels of *HMGCR*, *PPAR*-γ and *SCD-1* in the livers of the FBT-L, FBT-M, FBT-H, and ARA groups were significantly decreased (*P* < 0.01). In addition, compared with the expression in the control group, the mRNA expression levels of *HMGCR*, *PPAR*-γ and *SCD-1* in the cecum of the model group were significantly increased (*P* < 0.01), and the mRNA expression levels of *HMGCR*, *PPAR*-γ and *SCD-1* in the cecum of the FBT-L, FBT-M, FBT-H, and ARA groups were significantly decreased (*P* < 0.01), as shown in Figure 8. These findings indicate that FBT can improve lipid metabolism in the intestine and liver of NAFLD mice.

### 3.10 Correlation analysis between the gut microbiota, liver metabolites, and physiological data

**FIGURE 9:**
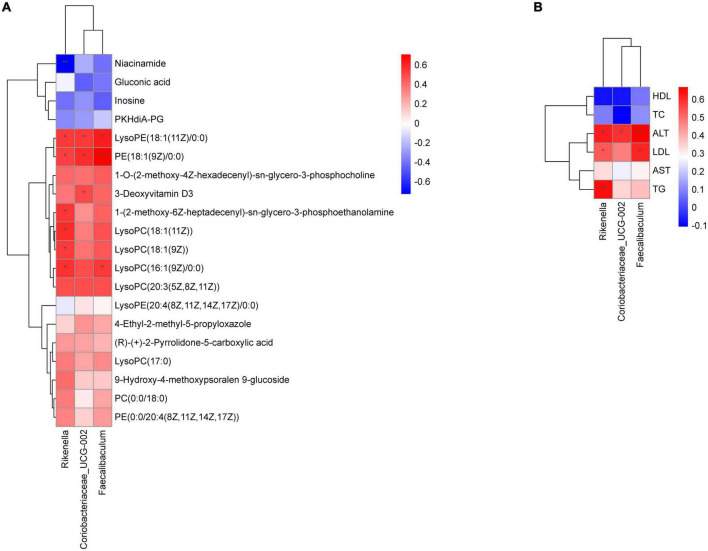
Correlation analysis of physiological data, liver metabolomics and gut microbiota. **(A)** Heatmap of gut microbiota constituents and differentially abundant metabolites; **(B)** heatmap of gut microbiota constituents and physiological data. *X*-axis represents the gut microbiota with differential abundance. *Y*-axis represents the differential metabolites **(A)** and physiological indexes **(B)**. Grids in red indicate positive correlations (correlation analysis value more than 0.1), while grids in blue indicate negative correlations (correlation analysis value less than –0.1), *p < 0.05, **p < 0.01.

Through the Spearman correlation coefficient, we found a close relationship between a variety of gut microbiota at the genus level between liver metabolites and physiological data. As shown in Figure 9A, *Rikenella* was positively correlated with various phospholipids (*P* < 0.05), and negatively correlated with niacinamide (*P* < 0.01). *Coriobacteriaceae_UCG-002* and *Faecalibaculum* were positively correlated with various phospholipids (*P* < 0.05). Additionally, *Rikenella* was positively correlated with TG, ALT and LDL (*P* < 0.01 or 0.05), *Coriobacteriaceae_UCG-002* was positively correlated with ALT (*P* < 0.05), and *Faecalibaculum* was positively correlated with ALT and LDL (*P* < 0.01 or 0.05), as shown in Figure 9B.

## 4 Discussion

At present, some studies have demonstrated the ability of FBT to alleviate high-fat induced obesity, insulin resistance and inflammatory diseases (15, 23, 24). However, the protective effect of FBT on high-fat-induced NAFLD is still unclear. In this study, we found that FBT can alleviate high-fat-induced NAFLD *via* the gut microbiota–liver axis. These results were comprehensively evaluated by metabolomics, analysis of gut microbiota analysis, pathological detection, biochemical detection and RT–qPCR (Graphical abstract).

The ideal animal model is a crucial factor in the study of the NAFLD. The impaired clearance of chylomicrons, LDL, very-low-density lipoprotein (VLDL) and other substances in the plasma of ApoE^–/–^ mice leads to the deposition of a large number of lipid substances in the liver. ApoE^–/–^ mice can better simulate the occurrence and development of human NAFLD, and are a reliable model for the study of NAFLD (12, 13). In this study, the body weight, liver weight, abdominal fat weight, and ALT and AST activities of the FBT groups were lower than those of the model group, which reflected that FBT played a protective role for the liver. The liver is an important place for the synthesis and metabolism of lipid substances. Excessive intake of lipid substances or metabolic disorders of lipid substances can lead to easy to deposition in the liver first, which then leads to the occurrence of fatty liver. Mild and moderate fatty liver can be reversed through diet adjustment or exercise. This study found that TC, TG and LDL in the serum of mice in the model group were significantly increased, and FBT could reverse the above changes, suggesting that FBT may promote the metabolism of lipids in the body or liver, thus effectively reversing the production of fatty liver. Further HE and oil red O staining showed that mice in the model group had a large amount of lipid deposits in the liver, and the intervention in the FBT-H and FBT-M groups was able to significantly reverse the deposition of lipids in the liver, but the FBT-L group was less effective in inhibiting lipid deposits, indicating that FBT must be given at a certain dose to effectively exert its effect in inhibiting lipid deposits. In addition, the inhibitory effect of the ARA group on the formation of lipid substances was worse than that of the FBT-H and FBT-M groups, which indicated that FBT could inhibit the deposition of lipids in a variety of ways.

The gut microbiota refers to the microorganisms that colonize the digestive tract and has the characteristics of large number, complex, dynamic, and diverse. Under normal circumstances, there is a dynamic balance between the flora and between the flora and the host. Once the balance is disturbed, a variety of diseases will occur (25, 26). The gut microbiota has important physiological functions, such as processing complex, non-digestible polysaccharides into short-chain fatty acids (SCFAs), providing energy for the host, and participating in the synthesis of bile acids and amino acids, immune regulation and maintenance of the intestinal mucosal barrier (27). Previous studies have shown that FBT can regulate the gut microbiota, increase the content of SCFAs in colon tissue, and improve lipid metabolism (28), suggesting that the gut microbiota may be a new target of FBT’s therapeutic mechanism. Our study showed that the structure and abundance of the gut microbiota in the model group were significantly different from those in the control group, and FBT could partially restore the composition and structural changes in the gut microbiota in the model group. In addition, we further found that FBT reversed the decrease in the ratio of *Firmicutes/Bacteroidetes* in the gut microbiota of mice in the model group and decreased the relative abundance of *Rikenella*. The ratio of *Firmicutes/Bacteroidetes* is often used to reflect the health of the gut microbiota and is closely related to steatosis and obesity (29). A large number of clinical studies have shown that the abundance of *Bacteroidete*s decreased and *Firmicutes* increased in the fecal microbiota from patients with NAFLD and cirrhosis (30, 31). The decreased abundance of *Rikenella* is beneficial to reduce body weight and promote cholesterol efflux (32, 33). Finally, we found that secretory and metabolic diseases, lipid metabolism and energy metabolism may be the main metabolic pathways of the different flora through PICRUSt2. Overall, our current results suggest that the inhibitory effect of FBT on NAFLD may be related to the regulation of the gut microbiota.

Anatomically, the intestine and liver are directly linked through the portal vein. Intestinal microbiota disorder will directly affect the liver and disrupt the metabolism of carbohydrates and lipids in the liver; that is, the occurrence and progression of NAFLD will be promoted through the “gut-liver-axis” (34). In addition, mammalian body fluid and tissue metabolomes are greatly influenced by the microbiota, and many health-related metabolites are considered “mammalian microbial cometabolites” (35). Based on the LC-MS metabolomics platform, we systematically analyzed the endogenous metabolites in the liver tissue of NAFLD mice treated with FBT and identified 64 differentially expressed metabolites as potential metabolic markers of the effect of FBT on NAFLD mice. These potential metabolic markers are mainly phosphatidylcholine (PC), lysophosphatidylcholine (LysoPC) and other metabolites closely related to lipid metabolism. Further enrichment analysis revealed that the regulation of adipocyte lipolysis and the sphingolipid signaling pathway may be the main metabolic pathways involved in FBT. The main function of adipocytes is to store excess fatty acids as a buffer, and dysregulated lipolysis is closely related to the occurrence of obesity, type 2 diabetes mellitus, fatty liver and other diseases (36). Sphingolipids are important components of biofilm structure and participate in many important signal transduction processes such as regulating cell growth, differentiation, senescence and programmed cell death (37). Recent studies have identified dysregulated sphingolipid metabolism in all stages of non-alcoholic fatty liver disease: steatosis, NAFLD, advanced fibrosis, and hepatocellular carcinoma (38, 39).

In addition, we studied the effect of FBT on the cecal barrier and factors related to lipid synthesis in the cecum and liver of NAFLD mice and found that FBT could ameliorate the cecal barrier and improve the mRNA expression of *HMGCR*, *PPAR*-γ and *SCD-1* in the cecum and liver of NAFLD mice. Tight junction proteins such as Occludin between intestinal epithelial cells are essential for the intestinal mucosal barrier to maintain the integrity of intercellular structures and ensure the normal function of their functions. Under normal circumstances, the structure of the gut microbiota is stable and in a relatively balanced state. Under such pathological conditions such as inflammatory stimulation and intestinal flora imbalance, the intestinal mucosal barrier is damaged, resulting in an increase in intestinal permeability. Intestinal pathogenic products may enter the blood circulation through the portal vein through the damaged intestinal mucosal barrier and cause a series of diseases. In this study, we found that FBT could improve the pathological morphology of the cecal and upregulate the expression of Occludin in NAFLD mice, suggesting that FBT could improve cecal pathology and cecal barrier in NAFLD mice. As a transcription factor whose metabolic enzyme activity is closely related to cholesterol metabolism, HMGCR can promote the synthesis of cholesterol, thus inducing the formation of fatty liver. Regulating HMGCR expression is a key step to maintain cholesterol homeostasis *in vivo* (40). PPAR-γ is closely related to the occurrence and outcome of obesity, diabetes, insulin resistance and other diseases (41). The expression of PPAR-γ is most abundant in the adipose tissue of animals (42) and can promote the deposition of free fatty acids in adipocytes. When hepatocytes exhibit steatosis induced by lipid deposition, the expression of PPAR-γ in the liver is significantly increased, and a large amount of PPAR-γ expression promotes the activation of lipid synthesis genes, leading to more lipid deposition in the liver (43). If PPAR-γ in hepatocytes can be removed, liver steatosis can be effectively avoided in mice (44). In conclusion, FBT may prevent and treat NAFLD by maintaining the homeostasis of the gut microbiota–liver axis homeostasis and inhibiting lipid synthesis.

Interestingly, the enrichment analysis of the gut microbiota and liver differential metabolites suggested that FBT may play a therapeutic role by affecting lipid metabolism. Therefore, we conducted a correlation analysis between the physiological data, liver metabolomics and gut microbiota. These results helped us to further reveal the potential mechanism of FBT in improving NAFLD through remodeling the gut microbiota, physiological data and liver metabolism. The results showed that *Rikenella* was positively correlated with various phospholipids, TG and ALT, and negatively correlated with Niacinamide. *Rikenella* is a sulfatase-secreting bacterium that induces bacterial endotoxemia and increased levels of chronic low-grade inflammation (45). Some studies have suggested that it may be a novel target for anti-diabetes and antioxidative stress (46). Moreover, *Faecalibaculum* was positively correlated with various ALT, LDL and various phospholipids. *Faecalibaculum* is considered to be a high-risk factor for obesity (47), and the regulatory mechanism may be associated with the regulation of intestinal epithelial Th17/Treg cell balance (48). In addition, *Coriobacteriaceae_UCG-002* was positively correlated with various ALT and various phospholipids. *Coriobacteriaceae_UCG-002* was demonstrated to be present in higher relative abundance in the gut microbiota of morbidly obese individuals and may have a potential dyslipidemic effect (49). Given these correlations, those likely the modulatory effects of FBT on liver lipid metabolism might occur through affecting the abundances of *Rikenella*, *Faecalibaculum* and *Coriobacteriaceae_UCG-002*.

However, this study has some limitations. We did not use germ free mice or fecal transplantation, and we could not determine which kind of microbiota is related to the therapeutic effect of FBT. In addition, the results of this study still need to be clinically verified, and the specific mechanism of differential microbiota or metabolites and lipid metabolism has not been thoroughly explored and needs further research. In addition, it is necessary to discover the role and mechanism of the relationship between FBT and intestinal epithelial cell permeability and SCFAs. Although further research is needed, according to the results presented here, FBT is expected to have therapeutic potential in NAFLD.

## 5 Conclusion

This study confirmed that FBT can alleviate high-fat-induced NAFLD. These findings suggest for the first time a possible mechanism by which FBT that FBT could regulate gut microecological dysbiosis, relieve intestinal barrier destruction, and regulate liver lipid metabolism, which, in turn, could ameliorate NAFLD.

## Data availability statement

The data presented in this study are deposited in the BioProject repository, accession number PRJNA858285 (https://www.ncbi.nlm.nih.gov/bioproject/PRJNA858285).

## Ethics statement

The animal study was reviewed and approved by the First Affiliated Hospital of Hunan University of Chinese Medicine (ZYFY20210710).

## Author contributions

YT and BC: conceptualization. XHu and XHe: methodology. JY: software. FT: validation. YL: formal analysis. YT: investigation, data curation, writing – review and editing, and funding acquisition. HZ: resources. BC: writing – original draft preparation. BL: supervision and project administration. All authors have read and agreed to the published version of the manuscript.

## Footnotes

1
https://www.gempharmatech.com

2
https://www.kegg.jp

## Supplementary material

Supplementary Material 1 The analysis protocol of metabolomics.

Supplementary Table 1 Differential metabolites between each group.

Supplementary Table 2 Differential metabolites of FBT reversed dysregulated metabolites.
